# Epigenetic Mechanisms in the Transcriptional Regulation of Circadian Rhythm in Mammals

**DOI:** 10.3390/biology14010042

**Published:** 2025-01-08

**Authors:** Wei Mao, Xingnan Ge, Qianping Chen, Jia-Da Li

**Affiliations:** 1 Department of Radiation Oncology, Zhejiang Cancer Hospital, Hangzhou 310000, China; maowei@ibmc.ac.cn (W.M.); gxn1106@163.com (X.G.); 2 Hangzhou Institute of Medicine (HIM), Chinese Academy of Sciences, Hangzhou 310000, China; 3 Center for Medical Genetics, School of Life Sciences, Central South University, Changsha 410078, China

**Keywords:** circadian rhythms, transcriptional regulation, histone modification, chromatin remodeling, Pol II pausing control

## Abstract

Almost all organisms, from the simplest bacteria to advanced mammals, have a near 24 h circadian rhythm. Circadian rhythms are highly conserved across different life forms and are regulated by circadian genes as well as by related transcription factors. Transcription factors are fundamental to circadian rhythms, influencing gene expression, behavior in plants and animals, and human diseases. This review examines the foundational research on transcriptional regulation of circadian rhythms, emphasizing histone modifications, chromatin remodeling, and Pol II pausing control. These studies have enhanced our understanding of transcriptional regulation within biological circadian rhythms and the importance of circadian biology in human health. Finally, we summarize the progress and challenges in these three areas of regulation to move the field forward.

## Abstract (plain language summary)

This literature review examines circadian rhythms, highlighting the crucial role of transcription factors in regulating gene expression, influencing plant and animal behavior, and affecting human disease. It examines the roles of basic transcriptional regulation in circadian rhythms, such as histone modifications, chromatin remodeling, and Pol II pausing control. The review also discusses further progress in the fine regulation of circadian rhythms and the importance of transcriptional regulation in circadian rhythms. This paper underscores the significant link between transcription factors and circadian rhythms, highlighting the latter’s contribution to improving human well-being.

## 1. Introduction

### 1.1. Circadian Rhythm

Circadian rhythms are internal biological processes following an approximately 24 h cycle, governing physiological and behavioral functions in response to light and darkness. These rhythms are regulated by a complex molecular clock consisting of core clock genes and their protein products, which interact in feedback loops to sustain rhythmicity. In mammals, the suprachiasmatic nucleus (SCN) in the hypothalamus serves as the main circadian clock, aligning physiological and behavioral functions like sleep–wake cycles, hormone release, and metabolism with the day–night cycle [1,2].

In 1971, Seymour Benzer and Ronald Konopka identified the genetic foundation of circadian rhythms by demonstrating that mutations in the Period (Per) gene in *Drosophila melanogaster* resulted in changes to these rhythms [3]. The PER protein is integral to the circadian clock, acting as a transcription factor in conjunction with TIM (Timeless) to control the expression of other circadian genes. This discovery provided a foundation for Jeffrey Hall, Michael Rosbash, and Michael Young to identify more circadian clock components and clarify the molecular mechanisms of circadian rhythms. Their work demonstrated that PER and CRY (Cryptochrome Circadian Regulator) form a negative feedback loop controlling the circadian cycle [4,5,6,7].

**Figure 1:**
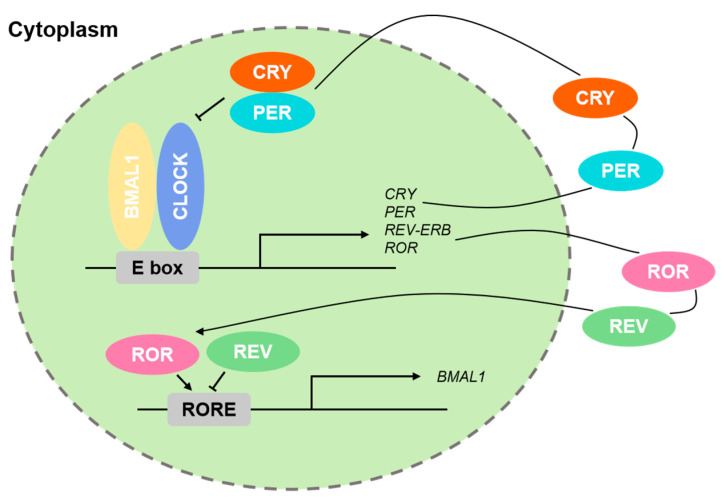
Core circadian rhythm loop in mammals. (1) Cry and Per Pathway: This is the primary feedback mechanism for the circadian clock. CLOCK and BMAL1 (activators) initiate the transcription of Per and Cry genes. The PER and CRY proteins then accumulate, form complexes, and translocate to the nucleus, where they inhibit the activity of CLOCK and BMAL1, thus shutting down their own transcription. This feedback loop is crucial for maintaining a roughly 24 h cycle. (2) ROR and REV Pathway: This pathway modulates the expression of clock genes, particularly *Bmal1*. ROR proteins (activators) bind to ROR response elements in the *Bmal1* gene promoter to enhance its expression, while REV-ERB proteins (repressors) bind to the same region to inhibit *Bmal1* transcription. The balance between ROR and REV-ERB activity helps stabilize the circadian rhythm and fine-tune its timing.

BMAL1 is a basic-helix–loop–helix (bHLH) transcription factor that heterodimerizes with the analogous bHLH transcription factor CLOCK [8]. The CLOCK–BMAL1 complex binds to E-box elements in circadian gene promoters, such as PER and CRY, to initiate their transcription [9]. This interaction is crucial for the maintenance of the circadian rhythm and is conserved across various species from fruit flies to mammals. In mammals, CRY1 and CRY2 proteins are crucial elements of the circadian feedback loop. They interact with PER proteins, causing their degradation and thereby regulating the feedback inhibition of CLOCK–BMAL1 activity [10]. The PER–TIM complex regulates CRY gene expression, contributing to the maintenance of the 24 h biological rhythm [11]. For a detailed summary of the expression and roles of these key proteins in the circadian rhythm, refer to Figure 1 and Table 1.

Since the discovery of the Key Components of Circadian Rhythms, many clock genes encoding transcription factors (TFs) have been cloned and characterized in mammals [15]. Despite significant progress, fundamental questions remain regarding how these molecules influence cellular autonomous rhythms from the transcriptional regulatory level to the physiological rhythms of the entire organism. Investigating how these molecules influence circadian transcription is essential for understanding organismal adaptation to circadian and other periodic environmental changes.

This review highlights recent advances in understanding the transcriptional mechanisms underlying circadian rhythms, particularly the influence of chromatin remodeling and protein modifications on transcriptional regulation, with a specific focus on RNA polymerase II (Pol II) pausing control. It is worth mentioning that by studying the endogenous rhythm mechanisms of organisms, we can apply this knowledge to adjust and optimize human lifestyle rhythms. Examining the impact of clock genes and biological rhythms on human health aids in developing informed lifestyle habits, enhancing physical and mental well-being.

### 1.2. Core Regulatory Circuits of Circadian Rhythms

Research on the regulatory mechanisms of circadian rhythms is crucial for understanding the operation principles of the biological clock, various activities of organisms, and the occurrence of diseases. We will describe the fundamental mechanisms regulating circadian rhythms in mammals, which underpin our comprehension of these biological cycles.

SCN in the hypothalamus regulates circadian rhythms in mammals by controlling gene expression, metabolism, and electrophysiological activities in response to light and other environmental cues [16,17,18]. The SCN’s biological clock structure is a transcriptional–translational negative feedback loop involving key proteins such as CLOCK and BMAL1, which bind to E-box elements in promoters to activate downstream gene expression. The expression of clock genes like *Period* (*Per1, Per2*), *Cryptochrome* (*CRY1, CRY2*), and *Rev-Erba* is regulated through negative feedback mechanisms. In this loop, PER and CRY proteins inhibit the CLOCK–BMAL1 complex by forming a heterodimer and translocating to the nucleus. CRY1 primarily lengthens the circadian period through strong and stable repression of CLOCK/BMAL1 activity, while CRY2 shortens the period with weaker and more transient repression. CRY1 is more stable and peaks earlier in the circadian cycle, whereas CRY2 is less stable and peaks later. Their complementary roles ensure balance and precision in the circadian clock. Additionally, *Rev-Erba* and *RORa* can respectively inhibit and promote the transcription of *Bmal1*, constituting another negative feedback regulatory loop [18,19,20]. For a detailed overview of these processes, refer to Figure 1 and Table 1.

Circadian rhythms are vital in plants, governing key physiological processes necessary for growth, development, and environmental adaptation. The circadian clock in plants regulates daily rhythms in photosynthesis, flowering, and hormone signaling, aligning internal processes with the external light–dark cycle [21]. Central to the plant circadian system are several core components and regulatory circuits that mirror those in mammals but also exhibit unique features adapted to plant life. Key elements of the plant circadian clock are TOC1 (TIMING OF CAB EXPRESSION 1), LHY (LATE ELONGATED HYPOCOTYL), and CCA1 (CIRCADIAN CLOCK ASSOCIATED 1). These proteins form a transcriptional feedback loop that regulates the expression of various clock-controlled genes. The circuit begins with the LHY and CCA1 proteins activating the TOC1 gene, which, in turn, represses the expression of LHY and CCA1. This negative feedback loop generates rhythmic changes in gene expression that drive daily physiological processes [22].

While the study of circadian rhythms in plants provides valuable insights into basic biological processes, this review will focus primarily on circadian rhythms in mammals. This emphasis is due to the profound implications of circadian rhythms on human health, as disruptions to these rhythms are linked to a range of diseases.

### 1.3. Circadian Rhythm and Diseases

Proper circadian function is essential for maintaining health, as disruptions to this delicate balance can lead to a variety of diseases (Table 2). Misalignment between internal circadian rhythms and external factors, like shift work, irregular sleep, or jet lag, is associated with metabolic disorders, cardiovascular diseases, and mental health issues. Research has shown that such circadian disruptions can lead to chronic conditions like obesity, diabetes, hypertension, and mood disorders by altering key physiological processes [23]. Understanding the impact of circadian rhythms on disease is an emerging research focus. This section explores how circadian disruptions contribute to disease pathology and highlights potential strategies to restore circadian balance for better health outcomes.

#### 1.3.1. Circadian Rhythm and Cancer

Circadian rhythm is closely associated with the occurrence of multiple cancers [24,25]. Environmental changes and hormonal disruptions can impair circadian rhythm genes, causing irregular cell cycles, inflammation, and carcinogenic signaling pathway activation, which heightens the risk of cancer cell proliferation and metastasis [26]. Irregular light exposure, including jet lag and shift work, is a potential risk factor for circadian disruption and cancer. Epidemiological studies indicate the association between circadian disruption and increased risk of specific types of cancer [27]. The breast cancer study revealed that disrupting the circadian rhythm markedly enhances cancer cell dissemination and lung metastasis in mice [28]. A study of 1900 Korean women identified a strong link between night shift work and a heightened risk of breast cancer, particularly among patients carrying the heterozygote genotype of *CRY2* rs2292912 or *RORA* rs1482057 [29]. Research on melanoma model mice exposed to chronic jet lag or continuous light exposure showed that circadian rhythm disruption promoted tumor growth by regulating immune responses and cell cycle regulatory factors [30].

#### 1.3.2. Circadian Rhythm and Metabolism

Circadian rhythm is crucial for maintaining metabolism, and mutation models of core clock genes reveal a close connection between circadian rhythm and metabolic dysregulation by perturbing immune responses and expression of cell cycle regulatory factors. *Bmal1* deficiency leads to circadian rhythm disruption, as well as weight gain, increased adipose tissue weight, and enlarged adipocytes, while also lowering the circulating levels of polyunsaturated fatty acids (PUFAs) [31]. Further research has found that pancreatic-specific *Bmal1* mutant mice showed impaired glucose tolerance and insulin secretion, along with elevated triglycerides and cholesterol levels, resulting in diabetes [32]. *Per2*- and *CRY*-deficient mice show alterations in lipid metabolism and gluconeogenesis [33,34]. Additionally, sleep disruption can lead to physiological and psychological stress, impair gut microbiota, and cause inflammation and metabolic diseases [35]. Studies have found that dysbiosis of gut microbiota in mice with disrupted circadian rhythms is affected by jet lag [36,37]. In summary, adjusting sleep and dietary patterns may be an effective approach to improving metabolic conditions.

#### 1.3.3. Circadian Rhythm and Neurodegenerative Diseases

Neurodegenerative diseases are closely related to circadian rhythms. Studies suggested that circadian disruption could be a risk factor for Alzheimer’s disease (AD) and Parkinson’s disease (PD), refer to Table 2. It was found that some circadian-related genes may be potential risk genes for AD. Changes in the rhythmic expression of *Bace2* and *ApoE* in the hippocampus of aged AD mouse models revealed circadian rhythms could impact AD [38]. Another survey study of 13 patients with AD found that they had longer resting intervals during the day, more frequent napping, and atypical circadian rhythms of melatonin release [39]. Research on patients with PD in China found that clock gene polymorphisms and circadian rhythm disruption were associated with the risk of PD [40]. These studies indicate that disrupting circadian rhythms may influence the development of neurodegenerative diseases.

#### 1.3.4. Circadian Rhythm and Neurodevelopmental Disorders

Circadian rhythm disruption is closely associated with neurodevelopmental disorders and sleep disturbances [41]. Autism spectrum disorder (ASD) shows a particularly significant relationship with circadian rhythm disruption, with up to 83% of patients with ASD exhibiting disruptions in sleep rhythms, leading ASD to be considered as a sleep disorder [42]. Recent research highlights notable disparities in sleep quality and circadian rhythm markers between adults with ASD experiencing sleep issues and healthy adults [43]. Research on monozygotic twins identified genetic and epigenetic factors in ASD, highlighting notable differences in gene methylation levels, especially increased methylation in the *RORα* gene promoter region in individuals with ASD compared to their healthy twin [44]. Related studies further emphasized the role of *RORα* in the occurrence of ASD and found that the RORα agonist SR1078 is associated with behavioral changes in ASD animal models [45]. A study identified significant associations between the circadian rhythm genes *PER1*, *NPAS2*, and ASD [46]. Research on circadian rhythm gene mutations has identified specific genetic alterations linked to ASD. In this study, 28 patients with ASD (14 with sleep disorders and 14 without) and 23 control subjects of Japanese descent were analyzed. These findings provide new clues for a deeper understanding of the pathogenesis of ASD [47]. These findings enhance comprehension of circadian rhythm regulation in neurodevelopmental disorders.

#### 1.3.5. Circadian Rhythm and Musculoskeletal Disorders

The circadian clock plays a crucial role in musculoskeletal health, with important implications for osteoarthritis (OA) and osteoporosis. Research indicates that circadian rhythm disruptions contribute to cartilage degeneration and OA pathology, with patients with OA showing circadian patterns in symptoms such as pain and stiffness, suggesting a link between circadian rhythms and joint health [48]. OA cartilage exhibits higher PER2 mRNA levels and reduced BMAL1 expression compared to healthy cartilage, indicating intrinsic circadian clock disruption in chondrocytes and associated increased MMP13 transcription and altered matrix composition [49]. Similarly, the dysregulation of circadian rhythm pathways in OA affects core clock genes and their networks, exacerbated by environmental factors like shift work, which worsen cartilage degradation in murine models [50]. In bone metabolism, disrupting Cry and Per genes in mouse osteoblasts increases bone mass, emphasizing circadian genes’ role in bone biology [51]. BMAL1, a central clock gene, regulates bone resorption and mass through osteoclast differentiation pathways. BMAL1 knockout mice exhibit elevated RANKL expression and osteoclast levels, leading to increased bone resorption [54], whereas BMAL1 overexpression reduces bone loss by modulating NFATc1 signaling [57]. Moreover, Per2 and Cry2 knockout studies highlight their roles in osteoblast and osteoclast functions, respectively, influencing bone formation rates and structure [52,53]. BMAL1 deficiency also impairs bone density and marrow differentiation, indicating its crucial role in maintaining bone integrity [55,56]. Therapeutic strategies targeting circadian mechanisms could offer new approaches for managing OA, osteoporosis, and other musculoskeletal disorders.

#### 1.3.6. Circadian Rhythm and Cardiovascular Disorders (CVDs)

Circadian rhythms are crucial for cardiovascular health, and their disruptions are associated with negative impacts on heart function and disease progression [58]. Genetic mutations in clock genes such as Bmal1, Clock, and Npas2 in mouse models have been shown to lower mean arterial pressure and impair sympathoadrenal responses to stress, indicating their essential role in cardiovascular regulation [59]. Specifically, cardiomyocyte-specific Bmal1 knockout results in impaired glucose utilization, accelerated dilated cardiomyopathy, and decreased longevity, underscoring the gene’s significance in cardiac health [60]. Additionally, disruptions in clock genes influence the expression of insulin signaling components in cardiac tissues, highlighting the link between metabolic and cardiovascular regulation [61]. Studies involving light/dark cycle manipulations, circadian period mutations, and SCN lesions have consistently demonstrated the profound impact of circadian disruptions on cardiovascular and immune functions, mirroring human data on circadian misalignment [62]. Intrinsic circadian regulation is evident in the heart, as core clock component oscillations have been observed in rodent and human tissues, as well as in cultured cardiomyocytes [63]. Cardiomyocyte-specific Clock-mutant mice, which maintain an unaffected central circadian system, exhibit disrupted heart rate rhythms and significant reductions in heart rate compared to wild-type mice [12]. These studies indicate that the cardiomyocyte circadian clock governs crucial heart functions, such as non-oxidative fatty acid and glucose metabolism, fatty acid responsiveness, contractility, and overall cardiac performance [63]. In summary, the circadian clock is integral to regulating heart rate, metabolism, signal responsiveness, contractility, and cardiac growth and regeneration, with disruptions in these rhythms contributing to cardiovascular disease (Table 2).

### 1.4. Transcriptional Regulatory Basis of Circadian Rhythms

The regulation of the mammalian biological clock involves not only the core regulatory loop (Figure 1) but also various transcription factors that modulate the core loop, as well as different types of modifications [8,64]. Transcription factors contain core transcription factors such as BMAL1, CLOCK, PER, and CRY; acidic amino acid transcription factors such as *DBP*, *TEF,* and *HLF;* and basic leucine zipper (bZIP) transcription factors such as *NFIL3/E4BP4*. Circadian transcription factors control the pacing and regulation of the oscillators as well as the downstream outputs, which are governed by complex feedback loops and post-translational modifications [65,66]. These factors promote transcription by recognizing D-box sequences on promoters and enhancers through competitive binding. *NFIL3* also has a function of transcriptional repression. Core transcription factors and *NFIL3* display antiphase activities, leading to diurnal expression patterns of their target molecules, like RORγ, that are inversely related. The peak expression of RORγ, occurring when REV-ERBa expression is at its lowest, enhances the transcriptional expression of *BMAL1* [67]. This interaction is equally crucial for regulating circadian rhythms. The phosphorylation of BMAL1 and CLOCK influences their activity and stability, thereby impacting the circadian rhythm [65]. PER and CRY proteins undergo phosphorylation by casein kinase 1 (CK1), which is important for their degradation and the rhythmic timing of the negative feedback loop [68].

In addition, multiple clock transcription factors have been found to share common binding sites on clock genes, indicating the existence of cooperative regulation by multiple clock transcription factors. Core transcription factors do not drive all core clock genes. Recent studies discovered a new circadian transcription factor, *CHRONO*, which exhibited circadian expression and inhibited *Bmal1* target genes by interacting with BMAL1–CLOCK and PER proteins [69,70,71]. Genome-wide studies have also revealed the widespread presence of post-transcriptional regulation, and most rhythmically expressed mRNA transcription molecules themselves do not have potential transcription rhythms. This means that, although many mRNA molecules are expressed rhythmically (i.e., their expression levels fluctuate in a 24 h cycle), their transcription process does not show a clear rhythmic pattern. Instead, the regulation of mRNA stability and degradation may play a crucial role in the circadian regulation mechanism [72,73,74,75,76]. mRNA processing elements like polyadenylation, histone modifications, and chromatin remodeling also influence the daily patterns of transcript accumulation and degradation [77,78].

**Figure 2:**
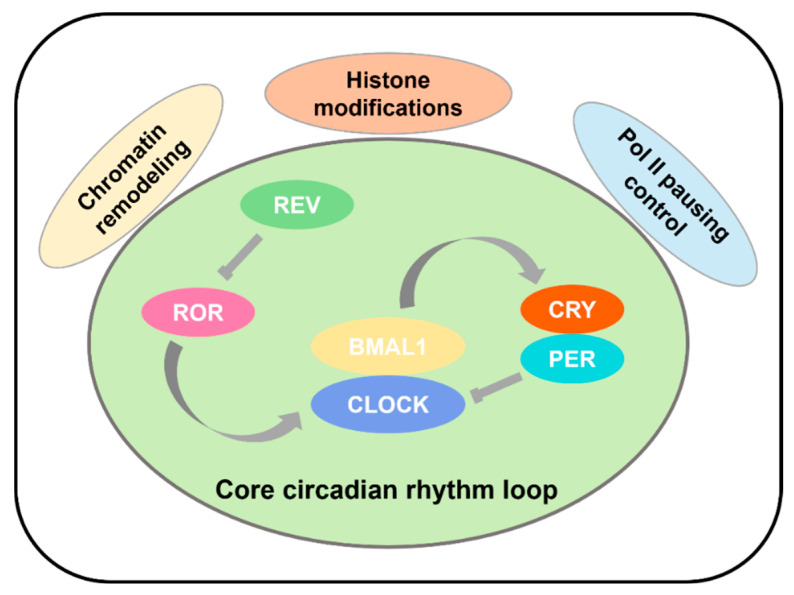
Illustration of negative feedback regulation in the circadian clock. Three key processes regulating transcription of the core circadian rhythm loop genes: histone modifications, chromatin remodeling, and Pol II pausing control.

Recent studies indicate that in complex systems, RNA Pol II is loaded onto promoters to initiate gene transcription, alongside active marks like H3K4me3 and H3K27Ac [79,80]. The reoccupation of these sites by PER1/2 and CRY2 transitions the process from promotion to inhibition, with subsequent delayed binding of CRY1 finalizing the loop reset (Figure 1). This review primarily focuses on histone modifications, chromatin remodeling, and Pol II pausing control outside the core transcription factors of circadian rhythms (Figure 2).

## 2. Circadian Rhythm and Histone Modifications

### 2.1. Histone Methylation

While DNA methylation levels in promoters show little variation over the circadian cycle, other epigenetic modifications, particularly histone methylation, exhibit dynamic oscillations that are tightly coupled with transcriptional rhythms [81]. Circadian rhythm genes directly influence histone modification regulatory factors, which reciprocally interact with the circadian rhythm, establishing a mutual regulatory mechanism [82,83,84]. Histone methylation is controlled by numerous histone methyltransferases (HMTs) and demethylases [85]. Histone methylation can correlate with transcription factor (TF) activity or inactivity. Histone basic residues can undergo mono-, di-, or trimethylation, each influencing transcription distinctly. Histone H3 lysine 4 trimethylation (H3K4me3) correlates with active promoters, whereas H3K4me1 is linked to enhancers [86,87,88]. H3K27me3, catalyzed by polycomb repressive complexes 2 (PRC2), acts as a repressive mark by serving as a scaffold for heterochromatin protein 1 (HP1), which induces nucleosome condensation and promotes a compact, transcriptionally silent heterochromatin state [89,90]. For example, PER facilitates the recruitment of the HP1γ–Suv39h HMT complex, which catalyzes the dimethylation and trimethylation of unmodified H3K9 residues, thereby promoting local chromatin repression [91].

### 2.2. Histone Acetylation

Histone acetylation is regulated by the interaction between histone acetyltransferases (HATs) and histone deacetylases (HDACs). Histone H3 acetylation at lysines 9 and 27 (H3K9ac and H3K27ac) enhances transcription factor binding, thereby activating transcription and increasing chromatin accessibility chromatin accessibility [92,93]. The bromodomain and extra-terminal (BET) protein family recognizes histone acetylation and is involved in transcription processes, including nucleosome remodeling and the recruitment of elongation factors [94,95]. Nuclear receptor corepressors (NCORs) interact with a range of factors mediating transcriptional repression, including HDAC3 [96]. HDAC3 recognizes the deacetylase-activating domain (DAD) of highly conserved NCOR1/2, which is essential for HDAC3 catalytic activity [97,98,99]. REV-ERBs recruit NCOR and HDAC3, collectively promoting histone deacetylation and repression of clock genes [100,101,102]. Subsequent studies indicated that deleting NCOR1 or HDAC3 specifically in the liver suppressed the expression of BMAL1 [100,101,103]. Additionally, mice with point mutations in the DAD of NCOR1 and SMRT (NS-DADm) show diminished HDAC3 recruitment and deacetylase activity, leading to altered clock gene expression and circadian behavior disruptions [104,105].

The intracellular redox state of NAD^+^/NADH also influences the circadian rhythm. The reduced forms of NAD(H) and NADP(H) can enhance DNA binding by directly binding to the BMAL1/CLOCK and NPAS2 heterodimers. Conversely, the oxidized variants, NAD^+^ and NADP^+^, mitigate this impact [106]. NAD^+^ acts as a cofactor for SIRT1, which rhythmically binds to CLOCK–BMAL1 and deacetylates PER2 and BMAL1 [107,108]. Deacetylation enhances PER2 degradation and reverses CLOCK-mediated acetylation of BMAL1, facilitating its interaction with CRY1 [108,109]. Interestingly, HDAC3 interacts with nuclear membrane proteins and the transcriptional repressor LAP2β independently of its deacetylase activity, anchoring chromatin to specific locations and inhibiting transcription [110,111,112]. NAD^+^ oscillations regulate SIRT1’s daily activity, forming a metabolic feedback loop linked to circadian transcriptional mechanisms. Additionally, AMP-dependent protein kinase (AMPK) increases the intracellular NAD^+^/NADH ratio during fasting, which activates SIRT1 to deacetylate and enhance the transcriptional activity of PGC1α [113,114]. Intracellular glucose can affect the activity of clock TFs through O-GlcNAcylation, while PER inhibits phosphorylation and enhances its own inhibitory activity through O-GlcNAcylation catalyzed by serine residues via O-GlcNAc transferase [115,116]. Furthermore, BMAL1 and CLOCK undergo rhythmic O-GlcNAcylation, which enhances the transcriptional activity of this heterodimer by competitively inhibiting its ubiquitin-mediated degradation [117].

### 2.3. Histone Phosphorylation

In mammals, the core transcriptional loop of the circadian rhythm triggers the expression of PER and CRY, which form heterodimers in the cytoplasm and are transported to the nucleus following phosphorylation by casein kinase I [10,68,118,119]. In the nucleus, the BMAL–CLOCK heterodimer interacts with lysine-specific demethylase 1 (LSD1) and JARID1A. Protein kinase Cα phosphorylates LSD1, enhancing its interaction with BMAL1–CLOCK to activate transcription [120]. Furthermore, phosphorylation processes, essential for metabolic homeostasis, regulate the circadian rhythm, with numerous phosphorylation pathways exhibiting feedback mechanisms to modulate this rhythm. Clock transcription factors can directly engage with metabolic pathway signaling elements, such as AMP-activated protein kinase (AMPK), a key regulator of metabolic signaling [121,122]. During fasting, AMPK is phosphorylated and activated by upstream signals, increasing the ratio of AMP to ATP [123]. Activated AMPK phosphorylates numerous proteins, including CRY1 in the liver. Phosphorylation of CRY1 leads to protein inactivation, degradation, and phase shifts in rhythmic expression in the liver [124]. AMPK also phosphorylates and degrades PER2 to regulate peripheral clocks by activating casein kinase I [125].

### 2.4. Histone Adenylation and Ubiquitination

Polyadenylation can influence the daily patterns of transcript accumulation and degradation [77,78,126]. Additionally, ubiquitination also influences the circadian rhythm loop. The reduction in CRY1 levels, which may activate loop transcription, could result from degradation facilitated by the E3 ubiquitin ligase FBXL3 [127,128,129,130]. These studies highlight the complex epigenetic regulation of circadian rhythms, detailing the interactions between clock transcription factors and co-regulatory elements and clarifying the roles of histone modifications and their epigenetic regulators in transcriptional processes. Some epigenetic regulatory factors may not necessarily require catalytic activity, challenging the concept of direct effects of histone modifications on transcriptional regulation, thus warranting further investigation [131,132,133]. Additionally, while an increasing number of histone modifications are being identified, their biological significance and transcriptional functions remain to be validated [134]. Therefore, it is imperative to elucidate the coordinated interactions, catalytic dependency, and independent functions of epigenetic regulatory factors in circadian transcriptional regulation.

### 2.5. Histone Sumoylation and Redox Modifications

Sumoylation, a post-translational modification, involves attaching small ubiquitin-like modifier (SUMO) proteins to target proteins, affecting their stability and function. SUMO modification of BMAL1 has been shown to affect its stability and transcriptional activity, thereby impacting the overall circadian rhythm [135,136]. Redox modifications, including cysteine residue oxidation, contribute to the regulation of circadian proteins [137]. The cellular redox state can affect CLOCK and BMAL1 activity, connecting circadian rhythms with metabolic and oxidative conditions [22,138].

## 3. Circadian Rhythm and Chromatin Remodeling

### 3.1. Chromatin Structure

In addition to histone remodeling, the circadian rhythm also regulates gene transcription rhythms by modulating chromatin structure. Nuclear architecture models suggest that chromosomes can form non-random aggregates, dynamically influencing metabolism, transcription processes, and development [139,140]. Therefore, changes in genome structure often occur in circadian rhythms. For example, chromatin conformation capture reveals rhythmic interactions between DBP and circadian loci on individual chromosomes, dependent on BMAL1 [141]. In mouse embryonic fibroblasts, PARP1 and CTCF regulate circadian rhythms by relocating clock-controlled genes (CCGs) to lamina-associated domains (LADs) [142]. Consistently, histone H3 lysine 9 methylation 2/3 (H3K9me2/3) consistently emerges as a crucial element in the repositioning of rhythmic genes to LADs [142]. Enhancer–promoter domains facilitated by cohesion are nested within larger CTCF–Cohesin domains [143]. *Rev-erba* suppresses *Bmal1* expression by recruiting nuclear receptor co-repressor (NCoR) and HDAC3 to suppress downstream expression [144]. These findings indicate that circadian rhythms regulate chromatin structure, with dynamic enhancer–promoter loop changes influencing the rhythmic expression of CCGs and potentially affecting various metabolic processes [145]. This raises crucial questions for future research, such as how rhythm-dependent chromatin structures and their dynamic changes affect cellular metabolic states.

### 3.2. Chromatin State

Chromatin, a DNA–protein complex, packages DNA within the cell nucleus, primarily arranged in repetitive arrays known as typical nucleosomes [146,147]. Translational modifications of chromatin serve as signaling factors regulating the accessibility or compaction of genomic elements. Simultaneously, accessibility or compaction is regulated by combinations of acetylation, phosphorylation, methylation, and ubiquitination, which can reshape chromatin states [148,149,150,151,152,153,154,155]. For example, from yeast to mammals, the conserved chromatin mechanism regulated by circadian rhythmicity (CRFH) forms and rhythmic molecules involving deacetylation, H3K9me, and HP1 binding engage in feedback inhibition.

The rhythmic post-translational modifications of chromatin can be explained by ATP-dependent remodeling enzymes and their equivalents. However, the specific modifications requiring remodeling enzymes lack a research foundation. ATP-dependent remodeling enzymes include conserved remodelers such as SWI/SNF (SWItch/sucrose nonfermentable) and the CHD family (CHD3/CHD4), both of which are necessary for the expression of circadian rhythm genes. In *Neurospora crassa*, the interaction between SWI/SNF and White Collar Complex (WCC) is necessary for the expression of the circadian gene FRQ (Frequency) [156]. Brahma (Brm), as the SWI/SNF complex in fruit flies, modifies the chromatin state of *dper* and *tim* to restrict Pol II recruitment. In mammals, the SWI/SNF subunit BAF60a plays a crucial role in liver circadian rhythms and is vital for energy metabolism [157]. Based on systematic studies, the SWI/SNF complex can alter the chromatin around transcription start sites to regulate the accessibility of Pol II and transcription factors [156,158]. Additionally, enhancer-associated lncRNAs, like lncCrot, play a role in regulating distal circadian enhancer interactions to enhance CCG expression [159]. However, the mechanisms by which lncRNAs, such as Per2AS, and eRNAs modify chromatin states to influence circadian rhythms remain unexplored and extend beyond the existing circadian regulation model.

### 3.3. Nucleosome Changes

The nucleosome is the basic structural unit of chromatin, consisting of four types of histones. Nucleosomes regulate circadian transcription, possibly because clock transcription factors (TFs) require tissue-specific TFs to bind to enhancers. These tissue-specific TFs interact with closed chromatin, reducing local nucleosome density. For example, the nuclear PER complex guides histone H3 lysine 9 methyltransferase (SUV39H1) to promote deacetylation and recruitment of the *Per1* and *Per2* promoters, leading to rhythmic di- and trimethylation of histone H3 lysine 9 (H3K9) [91,160].

In mammals, 30% of enhancer RNA (eRNA) is regulated by circadian rhythms, which is associated with nucleosomes H3K27ac and H3K4me1 [161]. CHD3 and CHD4 function as auxiliary repressive elements in circadian rhythm regulation within the nucleosome remodeling and deacetylase (NuRD) complex [162,163]. In *Neurospora crassa*, CSW-1 deficiency disrupts negative feedback in the circadian rhythm loop, facilitating nucleosome relocation to c-boxes and hindering White Collar Complex (WCC) binding [164]. The clock ATPase (CATP) promotes FRQ expression through c-box nucleosomes [165]. Overall, the presence of numerous nucleosome removal-related factors in circadian genes suggests the involvement of nucleosomes in circadian rhythm regulation. However, how these factors interact with modified nucleosomes and their exact catalytic activities remain unknown.

## 4. Circadian Rhythm and Pol II Pausing Control

Rhythmic proteins and transcription factors typically regulate the rhythmic transcription of gene promoters as distal enhancers. These transcription factors must engage with intermediate complexes and other transcription factors near the transcription start site (TSS) to regulate transcription. The recruitment and pausing of Pol II have recently received attention in the circadian field [8,79,166,167]. Pol II pausing, influenced by Pol II recruitment, pause release, and premature transcription termination near the TSS, is crucial for transcriptional output. Transcription factors regulate Pol II transcription at the start site by interacting with general transcription factors (GTFs) on the gene promoter through intermediate complexes [168,169]. The subunits of Pol II that interact with clock protein complexes remain to be determined. Transcription factors and co-factors guide intermediate complexes to assemble pre-initiation complexes (PICs) at the TSS for transcription initiation and re-initiation [170]. TFs and distal enhancers bound to promoters are believed to approach through chromatin looping, facilitated by proteins such as Cohesin and CTCF [171]. Transcription bursting involves multiple Pol II initiation events in a short time, regulated by enhancer–promoter proximity and recruitment of transcription factors and mediators, with underlying mechanisms still being explored [172]. Single-cell imaging studies show that transcription of numerous genes, such as clock genes, occurs randomly and infrequently [173]. Transcription within cells alternates between active and inactive states, characterized by transcription bursts followed by extended inactivity periods [174]. Transcription bursting can be explained by the formation of transcriptional centers, where TFs, co-factors, mediators, and Pol II aggregate/molecularly condense, facilitating multiple rounds of RNA polymerase II initiation [175,176]. Transcription bursting necessitates pause release, exemplified by p-TEFb-licensed Pol II elongation, which overcomes the +1 nucleosome barrier to transcribe the gene [177,178]. Once Pol II is initiated, it remains active for only a short period before entering a paused state, where it is held downstream of the transcription start site (TSS) by pause factors (DSIF and NELF) and the +1 nucleosome [179]. p-TEFb is a component of the super elongation complex (SEC), which facilitates paused Pol II, enabling it to re-initiate and complete transcription bursting [180,181]. Initiated Pol II could prematurely transcription termination at the 5′ end of the gene, reducing Pol II pausing [182,183].

The recruitment, pausing release, and premature transcription termination activities of Pol II exhibit genome-wide variations, peaking at distinct circadian phases [79]. During pausing release, p-TEFb recruits the PAF1 complex (PAF1c) to activate SET1, leading to the deposition of H3K4me3 downstream of gene TTSs [184,185,186]. Early in the night, H3K4me3 increases genome-wide in mouse liver [187,188]. [Pol II]_GB_ (Pol II signals in the gene body) shows more gene-specific changes throughout the day compared to [Pol II]_TSS_ (Pol II recruitment) and [Tbp]_TSS_ (Tbp signals within the transcription start site region) [126]. The bimodal distribution of gene transcription peaks aligns with the accumulation of pre-mRNA. Daily variations in the usage of [Tbp]_TSS_, [Pol II]_TSS_, and [Pol II]_GB_ could be related to the cell cycle, given its known impact on transcription [189]. In particular, transcription is typically suppressed during mitosis and reactivated at the end of mitosis [190,191]. Daily fluctuations in cell cycle activity interact with the circadian rhythm [192,193]. CDK1 activity, essential for mitotic entry, shows daily variations influenced by the circadian clock and reciprocally modulates clock oscillations [194,195].

**Figure 3:**
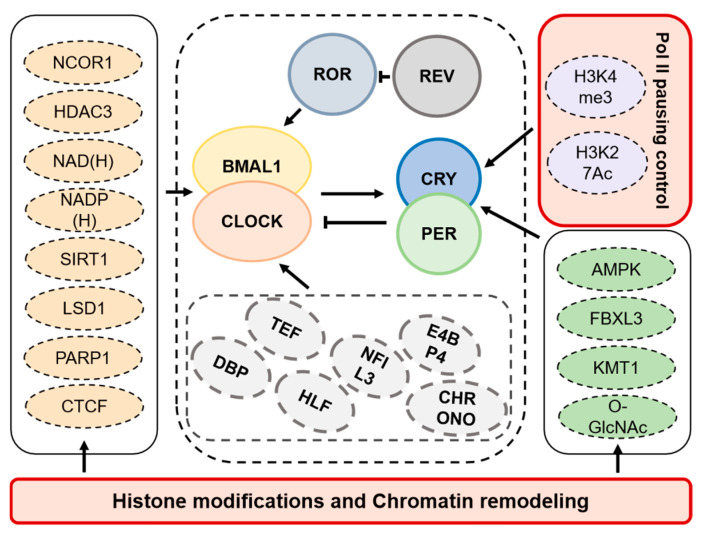
Transcriptional network involving core clock transcription factors and other regulators of the molecular clock. This figure illustrates the roles of key molecules in three primary transcriptional regulation pathways—histone modifications, chromatin remodeling, and RNA polymerase II pausing control. The core clock transcription factors interact with co-regulatory factors to orchestrate these pathways, influencing circadian rhythm regulation. Together, these processes establish a dynamic and coordinated regulation of circadian rhythms in mammals.

The recruitment of Pol II to the gene’s promoter does not directly influence transcription levels, challenging prevailing perspectives on Pol II recruitment regulation and transcriptional bursting [196]. However, this can be explained if pausing control is involved. If pausing release is necessary for transcriptional bursting and is independent of pre-initiation complex (PIC) regulation, then this paradox can be reconciled because transcriptional bursting occurs independently of PIC formation [197]. Recent research results indicate that when the mediator complex is rapidly degraded to restrict Pol II recruitment and initiation, there is a decrease in cell-type-specific gene transcription. Nevertheless, compensatory increases in pausing release help sustain the transcription of numerous other genes. These findings indicate that pausing release can affect transcription independently of Pol II recruitment (Figure 3 and Table 3).

## 5. Conclusions

This review investigates the transcriptional and epigenomic mechanisms through which circadian rhythm transcription factors control diurnal gene expression, highlighting the complexity of circadian rhythmicity in epigenetic regulation and questioning previous perspectives. We have discussed the new roles of chromatin structure, chromosome status, and nucleosome changes in circadian rhythm transcriptional regulation. These studies deepen our comprehension of circadian rhythm transcriptional architecture and inspire novel scientific questions and hypotheses. A major role of the circadian rhythm is to regulate chromatin state and structure, thereby influencing the timing and intensity of gene expression governed by circadian cycles. A conserved model incorporating deacetylation, H3K9me2/3, HP1 binding, and dynamic DNA methylation plays a crucial role in the negative feedback inhibition of circadian rhythms. Emerging connections between chromatin state changes and lncRNA are being identified. Finally, we have also highlighted the crucial role of Pol II pausing in circadian rhythm transcriptional output.

We highlighted how circadian rhythm transcription regulates metabolism through interacting mechanisms. Human genetics, epidemiology, and clinical research have associated circadian rhythm disruptions with various diseases and identified transcriptional regulatory mechanisms of circadian rhythms as fundamental for intervention studies. It further advances the molecular basis of circadian pathophysiology discovered at the systemic level. From transcriptional rhythms to circadian behavior, this will reveal more dimensions of the endogenous circadian rhythms of life on Earth. Circadian rhythm disruption has profound implications for human health, and in-depth research into the molecular mechanisms of circadian rhythm transcriptional regulation is crucial for identifying disease symptoms and mechanisms.

Although extensively studied, the exact mechanisms governing tissue-specific regulation of circadian rhythms are not yet fully understood. Distinct circadian gene expression patterns across tissues imply the existence of unknown regulatory elements and factors that contextually influence the core clock machinery. Understanding these tissue-specific regulatory networks is crucial for deciphering how circadian rhythms influence various physiological processes. The role of non-coding RNAs, such as microRNAs (miRNAs) and long non-coding RNAs (lncRNAs), in circadian regulation is gaining attention. Non-coding RNAs are crucial in modulating the expression of core clock genes and their downstream targets. The precise mechanisms through which non-coding RNAs affect circadian rhythms remain unclear, necessitating additional research to clarify their roles and regulatory networks.

Emerging evidence suggests a complex connection between circadian rhythms and metabolic processes, though the precise molecular mechanisms remain under investigation. For example, how do metabolic signals influence the core clock components, and conversely, how do circadian clocks regulate metabolic pathways? Exploring these questions may offer valuable insights for developing chronotherapy strategies for metabolic disorders. At the same time, while the influence of light–dark cycles on circadian rhythms is well established, the impact of other environmental and behavioral factors, such as diet, exercise, and social interactions, on circadian regulation is less well understood. Research in this area could identify novel external cues that modulate the circadian clock and uncover potential interventions for circadian-related disorders.

Addressing the questions mentioned above requires a multidisciplinary approach that combines molecular biology, genetics, bioinformatics, and systems biology. Advanced technologies like single-cell RNA sequencing, CRISPR gene editing, and high-resolution imaging are crucial for revealing the complexities of circadian regulation. Integrating circadian biology with neurobiology, endocrinology, and immunology offers a comprehensive understanding of the impact of circadian rhythms on health and disease. Collaborative efforts between researchers, clinicians, and industry will be essential to translate basic circadian research into therapeutic applications. Personalized chronotherapy, which considers an individual’s circadian profile, holds promise for optimizing treatment efficacy and minimizing side effects for various diseases. Advancements in circadian rhythm research will enable the development of new interventions and strategies to enhance human health and well-being.

## Figures and Tables

**Table 1:** Expression and roles of BMAL1, CLOCK, PER, and CRY in circadian rhythms.

Protein	Type	Role in Circadian Rhythm	Expression Locations	Conservation Across Species
BMAL1	Transcription factor (bHLH)	Forms a complex with CLOCK to drive circadian gene expression	SCN, liver, heart, and peripheral tissues [9]	Highly conserved across eukaryotes
CLOCK	Transcription factor (bHLH)	Forms a complex with BMAL1 to activate circadian genes	SCN, liver, brain, and peripheral tissues [12]	Highly conserved across eukaryotes
PER	Transcription factor (bHLH)	Represses CLOCK–BMAL1 activity in the feedback loop	SCN, liver, and various peripheral tissues [13]	Highly conserved from flies to mammals
CRY	Photoreceptor/Transcription factor	Binds PER to mediate degradation and regulate the feedback loop	SCN, retina, and peripheral tissues [14]	Highly conserved from flies to mammals

**Table 2:** Clock mechanism disturbance and diseases.

Method of Clock Disturbance	Effects	Type of Diseases	Data Sources	Impact	Refs
Environmental changes	Abnormal cell cycles, intracellular inflammation, activation of carcinogenic pathways	Multiple cancer	Human data	Whole-body	[24,25,26]
Irregular light exposure	Increased cancer cell proliferation and metastasis	BC, melanoma	Human data and mouse models	Whole-body and cell-specific	[27,28,29]
Shift work	Increased risk of breast cancer, particularly with certain genotypes	BC	Human data and mouse models	Whole-body	[27,28,29]
Chronic jet lag	Promoted tumor growth by regulating immune responses and cell cycle regulatory factors	Melanoma	Mouse models	Cell-specific	[30]
Bmal1 deficiency	Weight gain, increased adipose tissue weight, decreased glucose tolerance, reduced insulin secretion	Metabolic disorders, diabetes	Mouse models	Whole-body and cell-specific	[31,32]
Per2 and CRY deficiency	Alterations in lipid metabolism and gluconeogenesis	Metabolic disorders	Mouse models	Whole-body and cell-specific	[33,34]
Sleep disruption	Physiological and psychological stress, impaired gut microbiota, inflammation	Metabolic diseases	Mouse models	Whole-body	[35,36,37]
Circadian-related gene alterations	Potential risk genes for AD, altered rhythmic expression of specific genes	AD	Human data and mouse models	Whole-body and cell-specific	[38,39]
Clock gene polymorphisms	Associated with the risk of PD	PD	Human data	Whole-body	[40]
Circadian rhythm disruption	Increased frequency of napping, atypical circadian rhythms of melatonin release	AD	Human data	Whole-body	[39]
Circadian rhythm disruption	Significant disruptions in sleep rhythms, altered gene methylation levels	ASD	Human data and mouse models	Whole-body and cell-specific	[41,42,43,44,45,46,47]
Circadian rhythm gene mutations	Specific gene mutations associated with ASD	ASD	Human data	Whole-body	[46,47]
Intrinsic circadian clock disruption in chondrocytes	Increased MMP13 transcription, altered matrix composition	OA	Human data and mouse models	Whole-body and cell-specific	[48,49,50]
Cry and Per gene disruption in osteoblasts/knockout	Increased bone mass/Influenced bone formation rates and structure	Osteoporosis	Mouse models	Whole-body	[51,52,53]
BMAL1 knockout/deficiency	Elevated RANKL expression, increased bone resorption, impaired bone density/impaired bone density and marrow differentiation, maintained bone integrity	Osteoporosis	Mouse models	Whole-body	[54,55,56]
BMAL1 overexpression	Reduced bone loss by modulating NFATc1 signaling	Osteoporosis	Mouse models	Whole-body	[57]
Clock gene mutations	Lower mean arterial pressure, impaired sympathoadrenal responses to stress	CVD	Human data and mouse models	Whole-body	[58,59]
Cardiomyocyte-specific BMAL1 knockout	Impaired glucose utilization, accelerated dilated cardiomyopathy, reduced longevity	CVD	Mouse models	Whole-body	[60]
Light/dark cycle manipulations	Profound impact on cardiovascular and immune functions	CVD	Mouse models	Whole-body	[61]
Circadian period mutations	Disrupted heart rate rhythms, reduced heart rate	CVD	Human data and mouse models	Whole-body and cell-specific	[62]
SCN lesions	Mirrored human data on circadian misalignment effects	CVD	Human data and mouse models	Whole-body and cell-specific	[63]

**Table 3:** Overview of mammalian circadian rhythm transcriptional regulation.

Transcription Factors/Regulatory Genes	Description	Organisms Studied
CLOCK and BMAL1	Form a heterodimer that binds to E-box elements, promoting expression of downstream genes like Per1, Per2, CRY1, CRY2, and Rev-Erba.	Mammals
PERs and CRYs	Form heterodimers and inhibit BMAL1 transcriptional activity.	Mammals
Rev-Erba	Inhibits the transcription of Bmal1.	Mammals
RORa	Promotes the transcription of Bmal1.	Mammals
DBP, TEF, HLF	Acidic amino acid and basic leucine zipper transcription factors that recognize D-box sequences on promoters and enhancers.	Mammals
NFIL3/E4BP4	Recognizes D-box sequences and functions as a transcriptional repressor.	Mammals
CHRONO	Inhibits Bmal1 target genes by interacting with BMAL1–CLOCK and PER proteins.	Mammals
HMTs	Catalyze the methylation of histones, associated with transcriptional activation or repression.	Mammals
Histone demethylases	Remove methyl groups from histones, affecting transcriptional regulation.	Mammals
H3K4me3, H3K4me1	Marks associated with active promoters and enhancers, respectively.	Mammals
H3K27me3	A repressive mark catalyzed by PRC2, associated with transcriptional repression.	Mammals
H3K9me2/3	Associated with the repression of the chromatin state.	Mammals
HATs	Catalyze the acetylation of histones, promoting transcription and chromatin accessibility.	Mammals
HDACs	Removal of acetyl groups from histones leads to chromatin condensation and transcriptional repression.	Mammals
SIRT1	Deacetylates PER2 and BMAL1, influencing circadian rhythm.	Mammals
AMPK	Phosphorylates CRY1 and PER2, affecting their stability and activity.	Mammals
Pol II	Involved in transcription initiation, pausing, and elongation.	Mammals
SWI/SNF, CHD family	ATP-dependent chromatin remodeling enzymes are essential for circadian gene expression.	Mammals, *Neurospora crassa*, fruit flies
CTCF	Mediates chromatin interactions and regulation.	Mammals
PARP1	Mediates circadian regulation of chromatin by mobilizing clock-controlled genes to LADs.	Mammals
LncRNAs	Involved in regulating the interaction of distal enhancers to promote circadian gene expression.	Mammals
eRNAs	Enhancer RNAs involved in circadian regulation.	Mammals
Nuclear PER complex	Guides H3K9 methyltransferase to promote deacetylation and recruitment of Per1 and Per2 promoters.	Mammals
E3 ubiquitin ligase (FBXL3)	Mediates degradation of CRY1, influencing circadian loop activation.	Mammals
